# What are the roles involved in establishing and maintaining informational continuity of care *within *family practice? A systematic review

**DOI:** 10.1186/1471-2296-9-65

**Published:** 2008-12-09

**Authors:** Valorie A Crooks, Gina Agarwal

**Affiliations:** 1 Department of Geography, Simon Fraser University, 8888 University Drive, Burnaby, British Columbia, V5A 1S6, Canada; 2 Department of Family Medicine, McMaster University, 75 Frid Street, Hamilton, Ontario, L8P 4M3, Canada

## Abstract

**Background:**

Central to establishing continuity of care is the development of a relationship between doctor and patient/caregiver. Transfer of information between these parties facilitates the development of continuity in general; and specifically informational continuity of care. We conducted a systematic review of published literature to gain a better understanding of the roles that different parties – specifically doctors, patients, family caregivers, and technology – play in establishing and maintaining informational continuity of care within family practice.

**Methods:**

Relevant published articles were sought from five databases. Accepted articles were reviewed and appraised in a consistent way. Fifty-six articles were retained following title and abstract reviews. Of these, 28 were accepted for this review.

**Results:**

No articles focused explicitly on the roles involved in establishing or maintaining informational continuity of care within family practice. Most informational continuity of care literature focused on the transfer of information between settings and not at the first point of contact. Numerous roles were, however, were interpreted using the data extracted from reviewed articles. Doctors are responsible for record keeping, knowing patients' histories, recalling accumulated knowledge, and maintaining confidentiality. Patients are responsible for disclosing personal and health details, transferring information to other practitioners (including new family doctors), and establishing trust. Both are responsible for developing a relationship of trust. Technology is an important tool of informational continuity of care through holding important information, providing search functions, and providing a space for recorded information. There is a significant gap in our knowledge about the roles that family caregivers play.

**Conclusion:**

The number of roles identified and the interrelationships between them indicates that establishing and maintaining informational continuity of care within family practice is a complex and multifaceted process. This synthesis of roles provided serves as an important resource for continuity of care researchers in general, for the development of continuity of care quality indicators, and for the practice of family medicine.

## Background

Family medicine is a central component of comprehensive primary health care and a core principle in its delivery is the establishment and maintenance of continuity of care [1]. There is no single definition of continuity of care [2]; one way it can be characterized is through considering that it is comprised of interrelated dimensions, including: (1) informational (availability of recorded information); (2) longitudinal/geographical (having a regular site of care); and (3) relational/interpersonal (development of a trusting relationship between doctor and patient over time) [2,3]. Continuity of care is a hallmark of quality health service delivery [4] and a principle of planning in many primary care systems [5]. Characteristics attributed to continuity of care include: the availability of information in a medical record; the availability and/or constancy of a clinician; having a usual source or place of care; keeping follow-up appointments; providing seamless and coordinated care during transitions; and having an ongoing commitment to the patient [2]. Continuity is also understood to be an enabling factor and is positively correlated with having access to health services, achieving patient satisfaction, and decreased emergency department use [4].

As noted above, informational continuity is but one of several dimensions that contribute to continuity of care more broadly. This dimension of continuity serves as our focus for the remainder of this article. Informational continuity of care refers specifically to how well a patient's health information is able to 'travel' with him/her throughout the health service system, including over time, with the same practitioner and between practitioners in different settings [6]. Given this definition and our focus here on information exchange and recording within family practice, for the purpose of this article we consider information to be facts about or perceptions/experiences of symptoms, treatments, care management, health promoting strategies, and other focal points of family medicine practice that may be conveyed verbally, electronically, or in written form and exchanged between at least two individuals or recorded by an individual in some format (e.g. in a patient's file). Starfield's foundational primary care research identified the transfer of information across appointments to be an important attribute of continuity of care [2]. Information transfer across visits and between care providers is at the core of this dimension of continuity. Central to establishing informational continuity of care is the development of a relationship between doctor and patient/family caregiver that facilitates information transfer [2,5] (we use the term family caregiver to refer to unpaid/informal caregivers [i.e. non-experts] who escort individuals, typically family members, into appointments with doctors and are often expected to provide contextual information and/or to speak on behalf of the care recipient). There are also gains to certain patient groups, such as those managing chronic illnesses [5] and palliative care recipients [7], of experiencing informational continuity of care because of the nature of their care needs.

Informational continuity of care is a concept that is often used to describe information flow through primary, secondary, and tertiary care. However, we contend that establishing and maintaining informational continuity of care *within *family practice through information sharing between doctor and patient at the first point of contact is clearly important, particularly as the quality of such sharing can determine access to forms of care beyond the primary care setting through gatekeeping practices (e.g. to specialist referrals). In the remainder of this article we focus on the establishment and maintenance of informational continuity of care within a *particular *primary care relationship: between the family doctor and patient/caregiver as facilitated by technology. Because of the importance of continuity of care to the practice of family medicine, including for the treatment of episodic conditions, and because family doctors provide first-contact care and thus are centrally involved in establishing informational continuity across the continuum of care, family practice thus serves as our focus.

We conducted a systematic review in order to gain a better understanding of the roles different parties – namely doctors, patients, caregivers, and technology – play in developing informational continuity of care within family practice. Our purpose in undertaking this review is to synthesize the existing research on this topic in order to summarize our state of knowledge and also to identify gaps in our understanding. This review is both relevant and timely for several reasons, including that: (1) primary care reforms and restructuring that are resulting in patients increasingly being seen by multiple providers is making information sharing even more important [8,9]; (2) there are increasing numbers of chronically ill individuals [10] and palliative care recipients [11] throughout many countries and research has established that these (and other) groups have the most to gain from the presence of informational continuity [5,7]; and (3) such synthesis is useful not only to researchers but also those who work closely with patients. We sought out literature on the establishment of informational continuity of care within family practice, and specifically between patient/caregiver and doctor as mediated by technology. We report, in this article, on the findings of a systematic review conducted on this topic.

## Methods

A comprehensive, structured literature scan of published articles was performed for this systematic review. We focus in this article on a question used in the review pertaining to roles: What roles do patients, family caregivers, doctors, and technology play in establishing/maintaining informational continuity of care within family practice? Our inclusion of the 'roles of technology' comes in recognition of the fact that while computerized records are inanimate, they are central to information sharing within and beyond family practice and thus potentially have a role to play in establishing and maintaining informational continuity of care as a tool.

The electronic databases MEDLINE (OVID) (1966 – week 1, May, 2006), CINAHL (OVID) (1982 – week 1, May, 2006), EMBASE (1980 – week 1, May, 2006), PSYCHINFO (1806 – week 1, May, 2006), and Web of Science (1900 – week 1, May, 2006) were thoroughly searched. Articles in English pertaining to humans which were deemed relevant to the search were retrieved and managed using the Refworks bibliographic management program. All reference lists for the included articles were also hand searched for additional citations. We devised the search strategy by initially compiling keywords from broad literature searches on the electronic databases identified above. Search terms were refined through an iterative and collaborative process of reviewing outcomes of preliminary keyword searches in the databases. As shown in Table 1, the final search strategy included two intersecting areas of inquiry: (1) family medicine; and (2) informational continuity of care. Extra terms were added to focus the articles identified on the questions to be answered by the review.

**Table 1:** Overview of search terms and subjects

*Subject terms*	MeSH terms
*Family practice*	Family Practice/or family medicine.mp. or general practice.mp. or primary care.mp. or Primary Health Care/or general practitioner.mp. or Physicians, Family/or Primary Health Care/or Family Practice/or family practitioner.mp. or primary care physician.mp. or family physician.mp. or family doctor.mp. or Primary Health Care/or primary care clinician.mp. or Community Health Centers/or health care clinic.mp. or primary health care clinic.mp. or primary health care.mp. or family clinic.mp.

*Informational continuity of care*	"Continuity of Patient Care"/or informational continuity of care.mp. or Physician-Patient Relations/or medical records.mp. or Medical Records/or Medical Records Systems, Computerized/or written medical records.mp. or electronic medical records.mp. or Mental Recall/or Memory/or remembered information.mp. or knowledge.mp. or Knowledge/or Knowledge Bases/or notes.mp. or casenotes.mp. or Medical History Taking/or medical history.mp. or patient history.mp. or past medical history.mp. or Physician-Patient Relations/or Communication/or communication of information.mp. or patient charts.mp. or Medical Records Systems, Computerized/or patient records.mp. or patient medical history.mp. or doctor-patient communication.mp. or doctor-patient interaction.mp. or professional knowledge.mp. or physician-patient relations.mp. or Physician-Patient Relations/or Health Knowledge, Attitudes, Practice/or lay knowledge.mp.

*Establishment & maintenance*	Establish*; Maint*; Develop*

*Dimensions*	Dimension*; Aspect*; Level*

Titles and abstracts obtained from the initial searches were independently reviewed by both investigators. Articles of all types (e.g. research and conceptual) were included so as to be sure to obtain a comprehensive body of relevant literature. Articles focusing on issues outside of family medicine or primary care were excluded (i.e. we searched for references to general practice, family medicine, family doctors/practitioners, first-contact care, primary care), as were those which reviewed continuity of care in general or other types of continuity that did not explicitly mention or pertain to informational continuity of care. These were our only exclusion criteria in addition to limiting our scope to English-language articles, thereby excluding non-English publications. After independently selecting titles and abstracts, several meetings were held to discuss the inclusion or exclusion of all identified articles, with specific attention to those that the investigators disagreed about. Following this, full article review took place by both investigators. Upon reviewing all articles independently, the investigators held face-to-face meetings to discuss extracted information and confirm the inclusion status of each article.

Because of the nature of this review and the types of articles that were identified our focus was on systematically reviewing the *content *of included articles rather than the study design. A structured data extraction form was developed to ensure that the articles accepted for review were appraised in a consistent way by both investigators (see additional file 1 ). On the form information was recorded regarding the nature of the study design and the analytic technique employed. We primarily focused on extracting material from the articles pertaining to the questions guiding the review. Also recorded on the form was a listing of any references from the article to be followed up on and an indication of whether to accept or reject the article for the systematic review. All extracted information was then compiled into an electronic spreadsheet for use by the investigators.

Specific to the focus of this article, after compilation of the extracted data the investigators further reviewed all extracted information in the completed spreadsheet in order to delineate the roles specific to each group of focus. This was done in three steps. First, one investigator independently colour coded each piece of extracted data according to the group of focus (doctors, patients, caregivers, and technology). Second, the coded data was then confirmed with the other investigator. Third, based on the extracted coded information, roles pertaining to each group were interpreted and synthesized by one investigator and then confirmed by the other. There was full agreement between the investigators about the interpretation and synthesis of roles.

## Results

As shown in Table 2, 193 articles were obtained from all five bibliographic databases. A total of 56 articles were retained following the title and abstract review and identification of additional references from the bibliographies of reviewed articles. Twenty eight articles were ultimately accepted for this review. We excluded the other 28 reviewed articles because they did not meet our criteria for inclusion once the full papers were read and data were extracted. The kappa agreement scores for the 151 articles reviewed at the title/abstract stage was 0.673 (std. error of 0.17 and sig. of 0.004) which is reflective of a high level of agreement between investigators.

**Table 2:** Search strategy

**DETERMINATION OF SYSTEMATIC REVIEW QUESTIONS**	
↓	
**BROAD SEARCH OF LITERATURE TO DETERMINE KEYWORDS**	
↓	
**DETERMINATION OF KEYWORDS**	
↓	
**SEARCH 5 DATABASES USING KEYWORDS**	
↓	
**IDENTIFICATION OF ARTICLES FOR TITLE AND ABSTRACT REVIEW**	
193 articles source from 5 databases	
151 remain after removal of duplicates	
↓	
**INDEPENDENT REVIEW OF TITLES AND ABSTRACTS**	
↓	
**TITLE AND ABSTRACT REVIEW MEETINGS**	
112 excluded	
39 selected for full review (3 unobtainable)	
↓	
**INDEPENDENT ARTICLE REVIEW**	
↓	
**ARTICLE REVIEW MEETING 1**	
21 included	
15 excluded	
↓	
**INDEPENDENT ARTICLE REVIEW**	
20 new articles identified from references of reviewed papers	
↓	
**ARTICLE REVIEW MEETING 2**	
7 included	
13 excluded	
↓	
**OUTCOME**	
28 articles accepted for review	

Of the 28 articles that were included, three reported on mixed-method studies, six on qualitative studies, seven on quantitative studies, and the remaining 12 were commentary or review style articles. The work for three articles was undertaken in Canada, 11 in the United States, 11 in the United Kingdom, and one each in Australia, Norway, and the Netherlands. These articles are listed in additional file 2. In the remainder of the article we share the findings of the review by group (doctors, patients, and technology). Only one article referred to caregivers [14] and so we do not separately discuss this group.

In general, none of the reviewed articles focused explicitly on the roles involved in establishing or maintaining informational continuity of care within family practice. Because of this, in many cases roles needed to be interpreted by the investigators based on the extracted data. Most of the informational continuity of care literature reviewed focused on the transfer of information between settings and not at the first point of contact (i.e. within family practice).

### Doctors' Roles

Numerous articles contained implicit, and sometimes explicit, references to the roles doctors play in establishing and maintaining informational continuity of care within family practice. Central roles are to know how to effectively use the record keeping system [16,32] and to keep adequate records [25]. This is thought to be important so that they can review and recall recorded information [35] and deliver good quality care [16]. In addition to knowing how to enter information into the record, determining the information that gets recorded within it is another key role. This can include deciding whether or not non-medical (e.g. personal and social) information is included, which some suggest is important to the establishment of informational continuity of care [18,19,23,25]. The accuracy of the record as a whole can be determined by confirming information with patients during each visit [13], by regularly updating it [25], and even by developing strategies that allow patients to evaluate the information stored within [30].

Doctors serve as gatekeepers to the information that is shared and recorded within family practice in that they determine which pieces get shared with others [36]. They can also determine what information from other practices will be integrated into a patient's family practice record [15,22]. Delegating the updating of records to nursing or office staff is also within the remit of the doctor [14,33]. Because information shared within family practice as a result of the establishment of informational continuity of care may also be shared with other practices, other members of the same practice, or practice staff (e.g. administrative personnel), another role of the doctor is to ensure confidentiality [27,33]. This is very important to the information-sharing process as patients may be less willing to discuss personal details if they feel as though recorded information might be viewed by others [33]. At the same time, it is also the responsibility of the doctor to record information and not simply to rely on that which is remembered in the chance that the record needs to be accessed by someone else or the details are forgotten over time [16,17,22,23] despite confidentiality concerns.

### Patients' Roles

Patients also play many important roles in establishing informational continuity of care in family practice. Importantly, they need to establish trust in the doctor [23] – though certainly both doctor and patient play a role in trust building – and a willingness to share personal information, including socio-demographic details [16]. This includes being aware of what types of medical and non-medical details are important to share with clinicians [18]. While patients may hold views about the type(s) of information they want stored in their records, they need to make sure that such views do not result in holding back important details (e.g. personal, embarrassing in nature) that may be of use to the doctor at a later date [30].

Patients may benefit from playing an active role in managing and updating their medical records [30]. This can be done through keeping a summary of their health history that can be referred to when receiving acute care or switching family doctors [38]. As such, patients play an important role in developing and maintaining this kind of summary. Patients may also want to check that records are properly transferred between doctors, including when changing family practices [38]. Further, their remembered information may need to be drawn upon in order to reassemble or update the record [24].

Establishing a relationship with one's family doctor, particularly one that is long-term, is central to developing informational continuity of care in family practice [13,29]; this is also true for family caregivers involved in decision-making [14]. However, seeing the same family doctor each time may be out of the patient's control depending on the health care system and his/her individual resources (e.g. holding private insurance) [29]. Alternatively, patients may choose to see a new family doctor in order to get a fresh start with someone who is not overly familiar with their personal or medical history [17]. In such a situation the patient plays a great role in determining those elements of his/her history that will be shared with new doctors, particularly those not privy to their existing records.

### Roles of Technology

Computers and electronic medical records (EMRs) are an important element of information storage in family practice and thus play a role as a tool in establishing informational continuity of care. One reason for this is because computerized records are often more accurate than doctors' recollections or remembered information [34]. Information technologies in general enable timely and efficient patient-centred care [24], facilitate the development of longitudinal records [27], and generally enhance continuity [19,21]. Certain EMRs also have the ability to prompt doctors to take action and to be searched for health and demographic information [26].

EMRs can play a role in establishing informational continuity of care within family practice through providing space for historical and contextual information to be kept in the patient record [20]. Further, records of all forms (written or computer-based) can summarize the patient's history and previous care [28]. However, the use of the computers in general during appointments (e.g. for data entry into the EMR) can be perceived as disruptive, thereby negatively affecting or hindering the conversation or information sharing between parties and thus the ability to establish informational continuity of care [12].

## Discussion

Through interpreting the data extracted from the reviewed articles it is clear that there are many roles involved in establishing and maintaining informational continuity of care within family practice. These roles are summarized in Table 3. The number of roles identified and the interrelationships between them indicates that establishing and maintaining informational continuity of care within family practice is complex and multifaceted.

**Table 3:** Summary of extracted roles

Doctors' Roles	• knowing patient histories
	• using record keeping system(s) effectively
	• keeping adequate records
	• deciding whether personal/social information should be recorded
	• clarifying and updating record accuracy
	• determining what information gets shared with others
	• delegating updating of records to nursing or office staff
	• ensuring confidentiality
Patients' Roles	• transferring information to other practitioners
	• establishing trust in doctor
	• willingness to share personal information
	• withholding no important details
	• awareness of types of medical and non-medical
	• details that are salient
	• remembering important information

Roles of Technology	• serving as a tool to enhance continuity of care
	• prompting doctors to take action
	• being searchable
	• providing space for historical/contextual information
	• facilitating development of longitudinal record keeping
	• enabling timely and patient-centred care

### Limitations

Our comprehensive approach to scanning the existing literature and use of agreement between both investigators at all steps of the systematic review process are certainly strong points of our approach. A limitation is that we did not include a methodological assessment of the quality of reviewed articles. This was because the articles were too diverse for any objective scoring of quality that would be comparable across the board. However, because our focus was on reviewing the content of included articles to extract information about roles we do not believe that this limitation negatively affected our abilities to do this.

### Future Research

Our original intent was to include in this review an investigation into the roles that family caregivers play in establishing and maintaining informational continuity of care in family practice. This systematic review of the existing literature and our careful extraction of information from the selected articles revealed that there has been no consideration of this group and therefore it is a significant gap in our continuity of care knowledge. Our own exploratory research published elsewhere suggests that these individuals play an important role in determining topics of conversation and facilitating information transfer in general and must also work to establish a relationship with the doctor [39]. Thus, more research needs to be done in this area in order to more formally explore the roles of this group and their involvement in establishing and maintaining informational continuity of care within family practice.

The focus of our review has been on the roles involved in establishing informational continuity of care within family practice. After synthesizing this knowledge and identifying a key gap in our understanding of family caregivers' roles, we would be remiss in not suggesting research priorities. Research questions that warrant consideration include: what are the barriers to information sharing within family practice that could negatively affect informational continuity of care; what are the implications of not having informational continuity of care for all groups involved; how can we improve our understanding of informational continuity of care along with its role as part of quality care; and, lastly, what value do all parties in the information-sharing process place on the development and maintenance of informational continuity of care? We believe these research questions extend our present focus on the roles involved in establishing and maintaining informational continuity of care within family practice. Family caregivers, of course, should be included in such investigations.

In addition to the further research directions suggested above, there is also potential for additional knowledge synthesis as it relates to informational continuity of care. One possibility would be to extend the focus on roles to other provider groups both within and beyond primary care, including home care providers, allied health professionals, and specialists. Interactions these providers have with patients/caregivers are important to continuity of care in general, and specifically informational continuity of care as it exists outside of the family doctor-patient relationship that has been investigated in this systematic review. Another would be to investigate other bodies of literature, including the grey literature and policy and procedural documents that address issues of informational continuity of care or continuity more broadly. Such syntheses would greatly assist in further clarifying what is known about this topic while likely identifying additional knowledge gaps and thus avenues for new research.

### Implications

In general, this review reveals that while family doctors play an important part in extracting and configuring information about their patients, patients also play a significant part in establishing and maintaining informational continuity of care within family practice. Indeed, if patients are not engaged appropriately the chance for information-sharing will be lost. This is confirmed by Whiddett et al.'s [40] study of patient attitudes toward sharing health information. Further, it is well understood that the interpersonal relationship and communication style developed between doctor and patient is crucial to information-sharing processes such as diagnosis and disease management, among others [41,42]. These are clearly issues that warrant attention within the practice of family medicine in order to enhance the establishment and maintenance of informational continuity of care through the enactment of the roles identified in this systematic review.

The roles summarized in Table 3 are clearly relevant to numerous areas of clinical practice and its outcomes. Practice-based outcomes such as increased patient satisfaction [4,25,43] and improved care coordination [43] are acknowledged outcomes of continuity of care more generally. Given that informational continuity is a dimension of overall continuity of care, it is thus likely that undertaking strategies to ensure that one successfully undertakes the roles identified in this review could lead to these and/or other such outcomes. Based on the roles we have summarized, what strategies might be put in place to positively affect their enactment? The importance of the information recorded in the written or electronic record by the family doctor was a significant point of discussion in the reviewed articles. One strategy to improve this component of practice could be to ensure standardized recording system training, particularly within family practice groups or across practices using the same system. Through this process, EMRs in particular could be tailored to meet the informational needs of particular practice settings (e.g. in practices that see numerous patients with high-risk behaviours an EMR could prompt for this to be asked about and recorded) and the consistency of recording across practitioners could also be enhanced as an outcome. Furthermore, family doctors could openly discuss confidentiality with patients and caregivers and explain who will have access to their files as a way of encouraging information sharing. They could also discuss with patients and caregivers on an ongoing basis the types of information that they need in order to effectively provide care. Doing so could increase patients' awareness of the need to share details beyond experiences of specific symptoms and ultimately assist with creating a relationship of information-sharing.

Above we have cited particular strategies that could potentially be undertaken in order to enhance the enactment of the roles that doctors, patients/caregivers, and technology play in establishing and maintaining informational continuity of care. There are numerous other strategies that could be undertaken that are a logical extension of the systematic review findings to improve the outcomes of clinical practice. Strategies could also be tailored to the needs of particular patients; for example, certain trust-building-type practices could be put in place for facilitating two-way information exchange with patients reluctant to disclose personal details. Importantly, the effectiveness of any such strategies as they relate to practice-based outcomes, and possibly even health outcomes, would need to be evaluated before wide adoption.

Given the importance of having continuity of care, and specifically informational continuity, both within and beyond family practice, attention should be given to the ways in which specific care settings or practices facilitate its establishment. In this systematic review we have focused explicitly on the roles of particular groups. These roles, however, are undertaken within a larger context of care. Given the current climate of primary care reform (e.g. service restructuring, introduction of new technologies, shift towards providing care in the community [44]) that pervades many health care systems, opportunities for establishing continuity of care should be carefully considered. It is recognized, for example, that certain organizational features of some health care systems which can be the outcome of reforms, such as managed competition and increased reliance on urgent care/walk-in clinics, in fact undermine the establishment of such continuity [1,2,21]. This in turn threatens the very establishment, let alone ongoing maintenance, of informational continuity of care and prohibits the enactment of the roles identified in this systematic review.

## Conclusion

We asked: What roles do patients, family caregivers, doctors, and technology play in establishing/maintaining informational continuity of care within family practice? Our systematic review of the published literature has revealed that there has been little explicit consideration of such roles and that most literature is focused on the transfer of information across care settings. There are, however, numerous roles for doctors, patients, and information technologies in establishing and maintaining informational continuity of care within family medicine. There was almost no explicit consideration of caregivers' roles. The roles extracted from the literature and summarized in Table 3 are quite complex and require investment of time and resources to be effectively achieved. This synthesis of roles serves as an important resource for continuity of care researchers in general, for the development of continuity of care quality indicators, and for the practice of family medicine.

## Abbreviations

EMR: electronic medical record; GP: general practitioner.

## Competing interests

The authors declare that they have no competing interests.

## Authors' contributions

VC and GA played equal roles in the systematic review process. Each contributed to its design, read all selected abstracts/articles and extracted information for the database, and assisted with the analysis and interpretation of data. VC led the writing of this manuscript in that she identified its focus, gathered the relevant supporting materials from the review database, and led the writing/editing effort including developing the first draft. GA provided feedback at the outline stage, provided editorial comments throughout, and wrote part of the interpretation section.

## Pre-publication history

The pre-publication history for this paper can be accessed here:



## Supplementary Material

Additional file 1 **Data extraction form.** This is the extraction form that was created for this systematic review. Click here for file

Additional file 2 **Selected articles. **This is a detailed table summarizing the articles included in the systematic review. Click here for file
