# Supplementary material for: What are the roles involved in establishing and maintaining informational continuity of care within family practice? A systematic review
Source: BMC Fam Pract. 2008 Dec 9;9:65. doi: 10.1186/1471-2296-9-65 (PMC2626592; doi:10.1186/1471-2296-9-65)
Supplement: Additional file 1 — Data extraction form. This is the extraction form that was created for this systematic review. [file 1471-2296-9-65-S1.doc]

**Article-Extraction Sheet**

Date: ____________________ Reviewer:_________________________

| Title |  | | | | | | | | | | | | | | |
| --- | --- | --- | --- | --- | --- | --- | --- | --- | --- | --- | --- | --- | --- | --- | --- |
| Authors |  | | | | | | | | | | | | | | |
| Journal |  | | | | | | | | | | | | | | |
| Year |  | | | | | | | | | | | | | | |
| Country |  | | | | | | | | | | | | | | |
| Type of Study | Qualitative | | | | | | | Quantitative | | | | | Conceptual | | |
|  | Individual interviews | Focus groups | mixed | | Survey | | Other | Empirical /experimental | Cross-sectional | Time series | other | | editorial | letter | other |
| Sample | Sample size & target (specific professional group or patient diagnosis) | | | | | Demographics of population  Age_______________________________  Sex________________________________  Educational status____________________  Others____________________________ | | | | | | Sampling method (how sample was obtained) | | | |
| Analysis | Analytic technique/method used | | | | | | | | | | | | | | |
| Inclusion | - Include - Exclude | | | Reference Hand Search: | | | | | | | | | | | |

*Review Question:*

What is the role of patients in establishing/maintaining informational continuity of care in family practice?

What is the role of family caregivers in establishing/maintaining informational continuity of care in family practice?

What is the role of technology in establishing/maintaining informational continuity of care in family practice?

What is the role of the doctor in establishing/maintaining informational continuity of care in family practice?

________________________________________________________________________________________________________________________________________________________________________________________________________________________________________________________________________________________________________________________________________________

________________________________________________________________________________________________________________________________________________________________________________________________________________________________________________________________________________________________________________________________________________
