# Supplementary material for: What are the roles involved in establishing and maintaining informational continuity of care within family practice? A systematic review
Source: BMC Fam Pract. 2008 Dec 9;9:65. doi: 10.1186/1471-2296-9-65 (PMC2626592; doi:10.1186/1471-2296-9-65)
Supplement: Additional file 2 — Selected articles. This is a detailed table summarizing the articles included in the systematic review. [file 1471-2296-9-65-S2.doc]

**Appendix 2.** Selected articles

| **Author(s), Year Published** | **Study Design** | **Article Focus** | **Main Finding or Message** | **Summary of Extracted Information** |
| --- | --- | --- | --- | --- |
| Aaronson et al. [12], 2001 | qualitative survey of 219 family medicine residency directors | gather perceptions of family practice residents regarding using EMRs during residency | residents recognized the benefits of EMR systems but were frequently frustrated by them | - 41 % of respondents indicated that EMR use negatively affected the doctor-patient interaction. |
| Bertakis and Callahan [13], 1992 | cross-sectional observations of doctor-patient interactions in a university family practice; 47 interactions with established patients, 36 with new patients; interactions analysed using validated Davis Observation Code | explore differences in communication during visits between new and established patients | the nature of a visit is visit is dependent on the level of familiarity between doctor and patient | - Doctors may confirm/update information at each patient visit. - Patients who do not establish an ongoing relationship with a physician may never be targeted for health promotion efforts. |
| Burt et al. [7], 2004 | qualitative retrospective audit of GP communications from 13,460 patient consultations in palliative care situations | transfer of information between GPs and out-of-hour clinic providers in palliative care using information handover systems | attentiveness to and adoption of information transfer systems may assist with establishing continuity of care in the palliative care context | - Updating the information kept in patient records is an important role for doctors. |
| Desguin et al. [14], 1994 | conceptual (overview of practice guidelines) | presents guiding principles and management structures regarding primary care practice aimed at children with chronic conditions | providing paediatric care to children with chronic conditions requires careful attention to details such as the medical record | - Family is the primary source of continuity and care for chronically ill children. - Families need to receive information from primary care doctors to support-decision making. - Doctors may delegate the role of updating the medical records to office nurse staff. |
| Errington [15], 1974 | conceptual (essay) | presents the case of a particular patient, including social background | importance of the continuous nature of care in general practice must be recognized | - Doctors can request information on their patients from previous GPs - this is vital in order to make primary care effective. |
| Freeman [16], 1984 | conceptual (critical review) | presents a review and critique of continuity of care in general practice | many important gaps in our knowledge exist about continuity of care that should be investigated, including regarding the benefits of experiencing continuity | - GPs must maintain adequate records in order that good quality, consistent care can be delivered. - Informational continuity may be particularly important in those who are in extreme ends of life. - GPs may not have very good knowledge of the socio-demographic feature of their patients in the traditional model of care. |
| Freeman et al. [17], 2003 | conceptual (review) | presents an overview of how elements of continuity can be established in general practice in the new British system | gaining a greater understanding of continuity of care, particularly interpersonal, is essential for forwarding practice | - Sometimes both the doctor and patient want a fresh start/anonymity with regard to informational continuity. - A GP may deliberately not record contextual information so s/he becomes the keeper. |
| Freer [18], 1980 | conceptual (review) | presents a review of the use of health diaries in research | health diaries may have useful clinical applications in family medicine and provide the opportunity for patient-centred care | - Patients tend to record only medical problems in a health diary, not seeing psychosocial aspects as relevant. - Minor/transient health problems are often excluded from health diaries. - The more social aspects of patients’ lives are important for a holistic appreciation of health. |
| Guthrie and Wyke [19], 2000 | conceptual (short report) | presents a summary of organizational threats to continuity of care in general practice | GPs must ensure that organizational changes to practice have positive outcomes on their abilities to create personal continuity | - Personal continuity - ongoing doctor/patient relationship - ensures that care takes account of the patient’s personal and social context. - Continuity is enhanced by EMRs. - A chronically ill patient can spend up to 10 minutes explaining history to a new doctor - this may be all the time a doctor has. |
| Hamilton et al. [20], 2003 | cross-sectional retrospective case-control with cancer patients from 18 general practices | comparison of computer-only and paper-only recording systems | quality of the visit is highest in paper-only systems | - Some EMR systems have little 'free space' for recording important additional or contextual material. - If doctors do not record consultations somehow, then important follow/up information is lost. This problem is made worse if a different doctor takes the second consultation. |
| Hennen [21], 1975 | conceptual (essay) | presents an overview of the dimensions of continuity in family practice | there are 4 dimensions of continuity: chronological, geographical, interpersonal, and interdisciplinary | - Continuity of information is vital in cementing the inter-professional relationships in the office (as different professionals see patients). The medical record is the key to this. |
| Hjortdahl [22], 1992 | qualitative survey of 133 GPs after doctor-patient interaction | impact of doctors’ accumulated knowledge on decision-making | accumulated knowledge about the patient plays an important role in decision-making | - Lack of accumulated knowledge was a particular problem in patients presenting with psychological problems. - Prior knowledge about the patient affects decision-making in family practice. - Due to increasing specialization, GPs are becoming information coordinators. - Duration and depth of doctor-patient relationship, including trust, shapes accumulated knowledge (it takes at least a few years to establish a good knowledge base). |
| Hjortdahl [23], 2001 | conceptual (editorial) | presents a questioning of the continued relevance of continuity | balance between accessibility and continuity must be sought | - Continuity is a tool of family practice. - Informational continuity is not an adequate substitute for interpersonal continuity. - Consistent association between continuity and patient/doctor satisfaction. - Continuity builds the relationship between doctor and patient, the patient needs to trust the doctor. - Continuity of care is also used to develop the doctor's clinical knowledge and skills. |
| Kibbie et al. [24], 2004 | conceptual (editorial) | presents a discussion of the continuity of care record | the continuity of care record is an important part of the annual check-up | - Information technology enables safe, timely, efficient, equitable and patient-centered care. - A patient’s memory is sometimes needed when reassembling information. - Patients can relay electronic health records between physicians. - Patients have a legal right to access their own health information. |
| Liaw et al. [25], 1992 | two focus groups held with 21 randomly selected patients from family practice and walk in clinics | assess patient perceptions of continuity of care across socioeconomic groups | continuity of care should be thought of as an aid to consistent quality in family practice | - 10 patients indicated wanting to see a regular doctor because s/he would know their personal and medical history. - Patients assumed their doctors kept adequate records. |
| Litaker et al. [26], 2005 | survey of retrospective cohort of a specific patient group of 3718 patients (including a subgroup of 1448 patients with diabetes mellitus) | assess the contribution of continuity of care with a single doctor to short-term health outcomes where EMRs are used | continuity of care via single clinician likely has particular value in the management of established diseases that require care coordination between doctor and patient | - EMRs enhance information transfer and thus continuity. - EMRs can search for health and demographic characteristics and prompt physicians to take action. |
| Mandl et al. [27], 2001 | conceptual (review) | presents a review related to privacy in EMR use | in order to be most effective EMRs should be created according to public standards with patient input/control | - Technology facilitates the development of longitudinal (multi-site) medical records. - Most patients are not able to access their health data/medical records. |
| Moore and Busing [28], 1993 | qualitative survey of 13 family medicine residency program directors | gather information about how continuity of care is defined across residency programs, including how it is taught and evaluated | consensus regarding definitions of continuity of care is lacking but is needed to enable its teaching and evaluation | - There are many models of teaching and applying continuity of care in family practice. - Passive continuity = informational continuity. - Computer records summarize patient history and previous care. |
| Parchman et al. [29], 2002 | time series (cross-sectional prospective cohort) of 256 patients with type 2 diabetes | examine relationships between continuity, glucose control, and self-management | continuity of care (via single provider) results in better glucose control among type 2 diabetes patients | - Close-knit cultures are more likely to support doctor's orders. - Seeing the same provider is sometimes out of a patient's hands, due to financial changes. |
| Risdale and Hudd [30], 1997 | qualitative interviews with 39 patients from a specific clinic | explore patient views about what information they wanted to see recorded on computers during visits | doctors need to be attentive to how they communicate with patients when using computers during visits | - Patients have views about what information about themselves they see as being needed for storage on computer. - Lifestyle information and biological risk factors are appropriate types of information to be recorded. - Patients do not want personal comments to be visible to them, nor do they want serious illness to be recorded, unless discussed in advance. - Doctors need to develop ways by which patients can evaluate and access their information on computers. |
| Rogers and Curtis [31], 1980 | Conceptual (review) | presents an overview of the philosophy and definitions of continuity of care and proposes a model of continuity of care in primary care | not all dimensions of continuity can be measured, attention should be paid to modeling and evaluating measurable dimensions | - The patient is more likely to disclose personal information when s/he has an established record with the doctor. - Patients’ willingness to provide important contextual and health information is important for goal of creating continuity of care. |
| Rowan et al. [32], 2002 | cross-sectional survey of 134 family medicine preceptors using Primary Care Assessment Tool (PCAT) Provider Edition | determine how well preceptors provide quality care | preceptors provide quality care | - Coordinated medical records are important and enhance physician's abilities to recognize information about patients’ problems or therapies. Mechanisms to enhance this are problem lists, medical lists, and computers. - GPs may need computer training to enhance informational continuity. |
| Schers et al. [33], 2003 | cross-sectional postal survey of 873 family practice patients across 35 family medicine practices | explore patient views about access to information in their medical records by on-call doctors | assumptions regarding patient consent for access to their records should not be made | - Doubts about the confidentiality of the practice may lead patients to share information with their GPs less. - The increasing use of computers/proper record-keeping with EMR is useful for sharing/storing patient information. - The regular doctor is not necessarily the only person seeing records; there may be no control over confidentiality in larger practices due to turnover and assistants. |
| Starfield et al. [34], 1977 | random chart pulls of 200 patients | assessment of a medical record designed to enhance coordination of care | continuity is not the only factor that contributes to coordination | - Medical records are superior to doctors' recollections alone. |
| Starfield et al. [35], 1979 | observations of doctor-patient interactions and chart pulls of 104 patients with return visits scheduled | determine the extent to which the medical record contains evidence regarding coordination of care | the medical record reflects what information is deemed important by doctors | - Doctors routinely do not record certain types of information. - The quality of the medical record is linked to the quality of care. |
| The Bolton Research Group [36], 2000 | qualitative survey of 756 patients in 10 group practice clinics | investigate patient expectations regarding who should have access to their medical records | patients have clear expectations regarding access to their records | - Confidentiality allows patients to share information with doctors. - Patients believed that GPs should act as gatekeepers of the medical record. |
| Thompson [37], 1989 | conceptual (hypothesis statement) | presents a discussion regarding patient-held records among older patients | patient-held records could facilitate communication between providers | - Patients should have their own chart summary to use when travelling, switching doctors, and seeking acute care. - The doctor should establish a patient-based record so that when admission/intervention is needed all the information will be there. - Significant time can elapse between the new GP getting records in a transfer situation. |
| Toms [38], 1977 | multi-qualitative method and cross-sectional case study of 30 families who had lost their family doctors | Investigate the impact physician retirement on a specific practice | Patients can benefit from assistance in finding a new doctor in order to avoid some of the difficulties in transitioning to a new practice | - It may fall to the patient to ensure getting records transferred and getting continuity of care. |
